# Appearance of tolerance-induction and non-inflammatory SARS-CoV-2 spike-specific IgG4 antibodies after COVID-19 booster vaccinations

**DOI:** 10.3389/fimmu.2023.1309997

**Published:** 2023-12-20

**Authors:** Marjahan Akhtar, Md. Rashedul Islam, Fatema Khaton, Umma Hany Soltana, Syeda Anoushka Jafrin, Sadia Isfat Ara Rahman, Imam Tauheed, Tasnuva Ahmed, Ishtiakul Islam Khan, Afroza Akter, Zahid Hasan Khan, Md. Taufiqul Islam, Farhana Khanam, Prasanta Kumar Biswas, Faisal Ahmmed, Shakeel Ahmed, Md. Mamunur Rashid, Md. Zakir Hossain, Ahmed Nawsher Alam, A. S. M. Alamgir, Mahbubur Rahman, Edward T. Ryan, Jason B. Harris, Regina C. LaRocque, Meerjady Sabrina Flora, Fahima Chowdhury, Ashraful Islam Khan, Sayera Banu, Tahmina Shirin, Taufiqur Rahman Bhuiyan, Firdausi Qadri

**Affiliations:** ^1^Infectious Diseases Division, International Centre for Diarrhoeal Disease Research Bangladesh (icddr,b), Dhaka, Bangladesh; ^2^Bangladesh Institute of Tropical & Infectious Diseases, Chittagong, Bangladesh; ^3^Institute of Epidemiology, Disease Control and Research (IEDCR), Dhaka, Bangladesh; ^4^Division of Infectious Diseases, Massachusetts General Hospital, Boston, MA, United States; ^5^Department of Medicine, Harvard Medical School, Boston, MA, United States; ^6^Department of Immunology and Infectious Diseases, Harvard T.H. Chan School of Public Health, Boston, MA, United States; ^7^Department of Pediatrics, Harvard Medical School, Boston, MA, United States; ^8^Directorate General of Health Services, Dhaka, Bangladesh

**Keywords:** COVID-19, vaccine, booster, antibody, IgG, IgG4, tolerance

## Abstract

**Background:**

Understanding the characteristics of the humoral immune responses following COVID-19 vaccinations is crucial for refining vaccination strategies and predicting immune responses to emerging SARS-CoV-2 variants.

**Methods:**

A longitudinal analysis of SARS-CoV-2 spike receptor binding domain (RBD) specific IgG antibody responses, encompassing IgG subclasses IgG1, IgG2, IgG3, and IgG4 was performed. Participants received four mRNA vaccine doses (group 1; n=10) or two ChAdOx1 nCoV-19 and two mRNA booster doses (group 2; n=19) in Bangladesh over two years.

**Results:**

Findings demonstrate robust IgG responses after primary Covishield or mRNA doses; declining to baseline within six months. First mRNA booster restored and surpassed primary IgG responses but waned after six months. Surprisingly, a second mRNA booster did not increase IgG levels further. Comprehensive IgG subclass analysis showed primary Covishield/mRNA vaccination generated predominantly IgG1 responses with limited IgG2/IgG3, Remarkably, IgG4 responses exhibited a distinct pattern. IgG4 remained undetectable initially but increased extensively six months after the second mRNA dose, eventually replacing IgG1 after the 3rd/4th mRNA doses. Conversely, initial Covishield recipients lack IgG4, surged post-second mRNA booster. Notably, mRNA-vaccinated individuals displayed earlier, robust IgG4 levels post first mRNA booster versus Covishield counterparts. IgG1 to IgG4 ratios decreased with increasing doses, most pronounced with four mRNA doses. This study highlights IgG response kinetics, influenced by vaccine type and doses, impacting immunological tolerance and IgG4 induction, shaping future vaccination strategies.

**Conclusions:**

This study highlights the dynamics of IgG responses dependent on vaccine type and number of doses, leading to immunological tolerance and IgG4 induction, and shaping future vaccination strategies.

## 1 Introduction

COVID-19 vaccines have played a vital role in the global efforts to mitigate the transmission of the SARS-CoV-2 pandemic. In Bangladesh, nine COVID-19 vaccines have been approved for use (1) including messenger RNA (mRNA) vaccines BNT162b2 (Pfizer-BioNTech) and mRNA-1273 (Moderna); adenovirus-based vaccines ChAdOx1-S (Covishield, Serum Institute of India) etc. The effectiveness of COVID-19 vaccines has been a crucial determinant in reducing the burden on healthcare systems and preventing countless fatalities worldwide (2). A prospective, hospital-based, test-negative case-control study carried out in Bangladesh revealed that two full doses of the mRNA-1273 vaccine provided considerable protection compared to the ChAdOx1 nCoV-19 (3). Furthermore, Andrews et al. demonstrated the relative effectiveness of booster doses of BNT162b2 or mRNA-1273 vaccines against symptomatic disease in individuals initially administered either ChAdOx1-S or BNT162b2, ranging from 85% to 95% (4). Despite these noteworthy efficacy findings, it has been reported that vaccine-induced humoral immunity wanes over time, leading to a decrease in effectiveness after the administration of the second and third vaccine doses (5).

In Bangladesh, we have demonstrated that COVID-19 patients and individuals who have been fully vaccinated exhibit heightened levels of antibodies targeting the spike protein of the SARS-CoV-2 virus (6). Our group has shown that the administration of ChAdOx1 nCoV-19, mRNA-1273 and BNT162b2 vaccines resulted in a significant increase in spike receptor-binding domain (RBD)-specific antibodies following the initial two doses, followed by a subsequent decline after six months (6). Despite the ability of these vaccines to elicit significant anti-spike IgG responses, their protective effect against SARS-CoV-2 infection seems to be only temporary and not broad enough (7), as evidenced by the high incidence of breakthrough infections caused by the Omicron variant (8, 9). After COVID-19 infection and vaccination, notable modulation is observed among the four IgG antibody subclasses: IgG1, IgG2, IgG3, and IgG4. IgG1 and IgG3, predominant proinflammatory subclasses, are detected post SARS-CoV-2 infection and shortly after primary mRNA vaccination (10). These subclasses are linked to pathogen neutralization and effector mechanism activation. In contrast, the anti-inflammatory IgG4 subclasses are rarely found in the bloodstream and possess inhibitory effector functions due to their unique characteristics, such as being bi-specific and functionally univalent as a result of “Fab-arm exchange” (11). Elevated levels of IgG4 antibodies have been associated with fatal COVID-19 cases and has been identified as a predictor of mortality in COVID-19 cases (12, 13). Notably, mRNA vaccines stimulate IgG4 production, with three mRNA vaccine doses inducing prolonged IgG4 responses and potentially contributing to immune tolerance (10, 14).

Evaluating the dynamics and significance of IgG subclass responses following COVID-19 infection and vaccination is essential for assessing immune protection, monitoring vaccine efficacy, and designing future vaccination strategies. However, there is limited information on the dynamic levels of subclasses of IgG antibodies after two primary doses and two mix-match booster COVID-19 vaccination in Bangladesh. In this study, we have analyzed the longevity and kinetics of spike RBD-specific IgG antibodies and its subclasses at different time points after primary and booster COVID-19 vaccination in Bangladesh.

## 2 Methods and materials

### 2.1 Study design

The study was carried out in Bangladesh Between 2021 and 2023. Participants were recruited from Kurmitola General Hospital, Dhaka, Bangladesh. The study included individuals who had received four doses of COVID-19 vaccines including ChAdOx1 nCoV-19 (Covishield, Serum Institute of India), mRNA-1273 (Moderna), or BNT162b2 (Pfizer-BioNTech) as primary or booster doses between March 2021 and April 2023. The overall goal of this study was to evaluate the dynamics of SARS-CoV-2 spike RBD specific IgG subclass antibody responses in participants who received four COVID-19 vaccine doses. We have only considered participants who received mRNA vaccine as booster doses after receiving two primary mRNA doses or two primary Covishield doses. Participants were grouped based on primary vaccination regimen (dose 1 and dose 2). Group 1 received mRNA vaccines and group 2 received Covishield as primary vaccines. All participants in this study received mRNA vaccine as booster 1 (dose 3) and booster 2 (dose 4). Prior to enrollment and within visits, participants underwent interviews where information about their age, gender, history of prior COVID-19 infection, and co-morbidities was recorded. Three participants from each group had confirmed SARS-CoV-2 infection before receiving primary doses and one participant from each group had confirmed SARS-CoV-2 infection before receiving booster 1 (**Table 1**).

**Table 1:** Demographic information of the study participants.

	Group 1	Group 2	Total
Number of participants, n	10	19	29
Primary vaccines (Dose 1 and Dose 2)	mRNA	ChAdOx1-S (Covishield)	–
Booster vaccines (Dose 3 and Dose 4)	mRNA	mRNA	–
Covishield received as primary vaccines, n (%)	–	19 (100.0)	19 (65.5)
mRNA-1273 (Moderna) received as primary vaccines, n (%)	9 (90.0)	–	9 (31.0)
BNT162b2 (Pfizer-BioNTech) as primary vaccines, n (%)	1 (10.0)	–	1 (3.4)
mRNA-1273 (Moderna) received as booster vaccines, n (%)	6 (60.0)	–	6 (20.7)
BNT162b2 (Pfizer-BioNTech) as booster vaccines, n (%)	4 (40.0)	–	4 (13.8)
SARS-CoV-2 infection before primary doses, n (%)	3 (30.0)	3 (15.7)	6 (20.7)
SARS-CoV-2 infection before booster doses, n (%)	1 (10.0)	1 (5.3)	2 (6.9)
Age in years, median (range)	37.5 (30-53)	44 (29-60)	43 (29-60)
Sex, n (%)			
Female	4 (40)	6 (31.6)	10 (34.5)
Male	6 (60)	13 (68.4)	19 (65.5)
Time intervals between vaccination in days, median (range)			
Dose 1 to Dose 2	28 (28-29)	63 (54-76)	–
Dose 2 to Dose 3	235 (225-338)	302 (276-312)	–
Dose 3 to Dose 4	329 (246-371)	372 (362-388)	–

All participants provided informed written consent. The study was approved by the Institutional Review Committee of the International Centre for Diarrhoeal Disease Research, Bangladesh (icddr,b), and the Institute of Epidemiology, Disease Control and Research (IEDCR).

### 2.2 Specimen collection

Blood samples were obtained from participants at various time intervals: prior to the first dose of the vaccine (Pre-V), One/two months after the first dose which is before the second vaccine dose (1M), one month after the second dose which is 2/3 months after first vaccine dose (2/3M), and at six months (6M) after receiving the first primary COVID-19 vaccination. Blood samples were collected again one month after booster 1 (12-13 months after 1st primary dose) and booster 2 (24 months after 1st primary dose). After centrifuging the blood at 700×g for 15 minutes, the sera were separated and subsequently frozen at -20°C until the laboratory analysis.

### 2.3 Determination of RBD-specific IgG and IgG subclasses antibodies

RBD-specific IgG antibodies were determined using Enzyme-Linked Immunosorbent Assay (ELISA) as previously described (6, 15, 16). To determine IgG subclasses antibodies (IgG1, IgG2 IgG3 and IgG4), 100 µL of serum samples were added to the plate which were initially diluted to 1:10 and then 3-fold, 8 serial dilution was performed. Plates were subsequently incubated at 37°C for an hour. Mouse anti-human IgG1 (1:3000), IgG2 (1:4000), IgG3 (1:3000) and IgG4 (1:3000) Fc specific HRP (Southern Biotech) were added to the plates and incubated for one hour at room temperature. Subsequently, Ortho- phenylenediamine in 0.1 M sodium citrate buffer (100 µL; pH 4.5) and 30% hydrogen peroxide were added to the plate. The reactions were stopped after 20 minutes by adding 1 M H_2_SO_4_ (25 µL) and endpoint titers were determined as the reciprocal interpolated dilutions of the samples at 492 nm that were 0.4 above the background.

### 2.4 ELISA analysis of cytokine

The concentrations of IL-10 cytokine in serum specimens were determined using sandwich ELISA (eBioscience, USA) following the manufacturers’ instructions.

### 2.5 Statistical analyses

Antibody responses in vaccinated participants before and after vaccination were analyzed in this study. Statistical analysis was performed between pre- and post-vaccination using the Kruskal-Wallis test. Fold increase was calculated as follows: one-month post vaccination antibody concentration versus pre vaccination antibody concentration for each dose. Graph Pad Prism (version 6.0) was used for generating plots and analyses.

## 3 Results

### 3.1 Study participants

A total of 29 adults (≥18 years, Median age: 43 years; IQR 38-48 years) were included in this study who received four doses of COVID-19 vaccines in Dhaka city. Specifically, 10 adults received four doses of mRNA vaccines, comprising two primary doses and two booster doses. 19 adults received two primary doses of ChAdOx1 nCoV-19 (Covishield) followed by two booster doses of mRNA vaccines. Among the participants in the study, 65.5% (n=19) were male, while 34.5% (n=10) were female, as indicated in **Table 1**.

### 3.2 IgG responses after primary and booster COVID-19 vaccination

**Figure 1:**
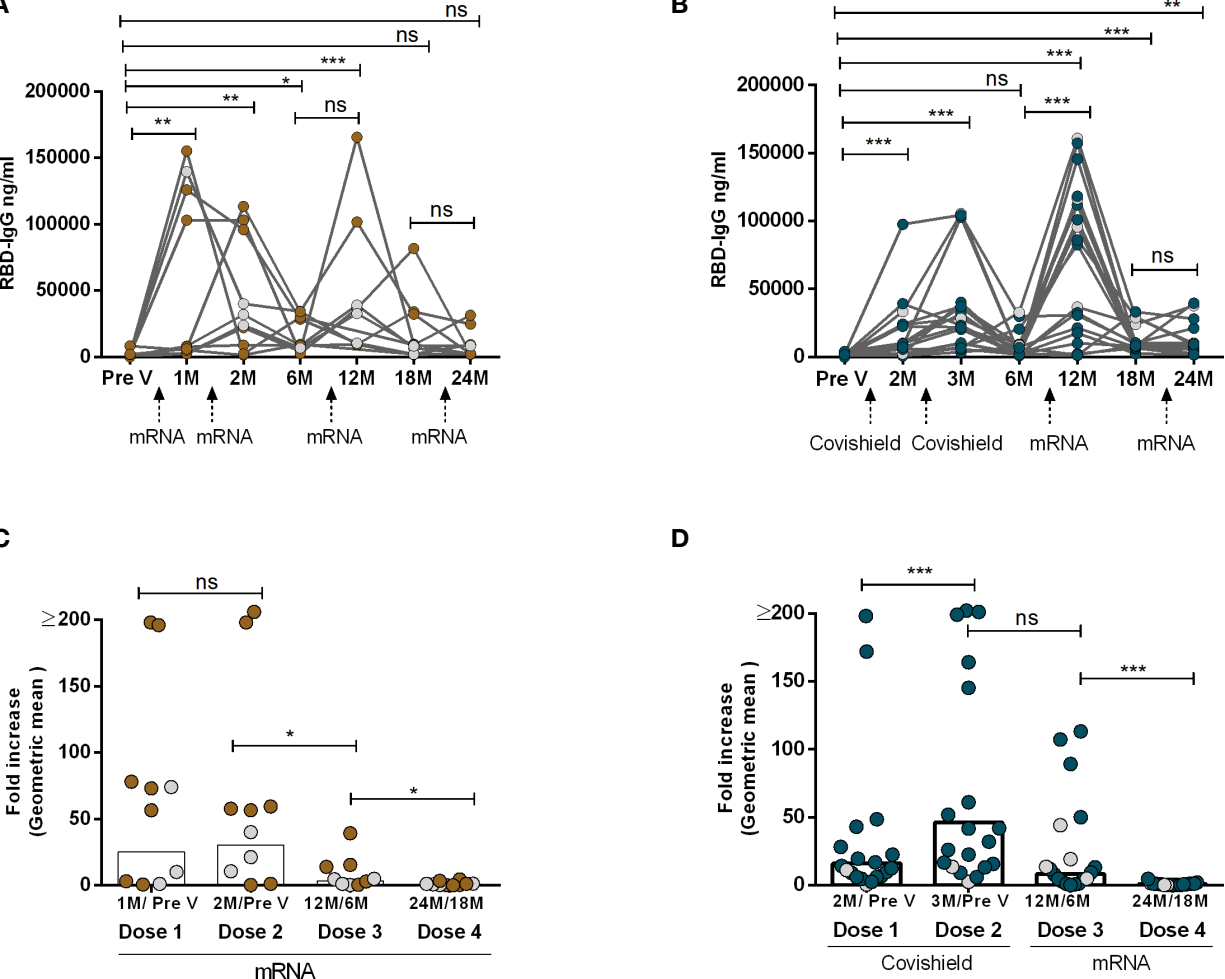
IgG Antibody responses after two primary and two booster COVID-19 vaccination. RBD-specific IgG was analyzed in serum samples after four mRNA doses **(A, C)** and two primary Covishield and two booster mRNA doses **(B, D)** at different time intervals. Different doses of vaccine administration time points are indicated below the graphs. Samples were collected before vaccination (Pre-V), 1 month, 2/3 Months, 6 months, 12 months, 18 months, and 24 months after the first vaccination. Fold increase was calculated 1 month post-vaccination IgG conc. vs pre-vaccination IgG conc. for each dose as indicated in Figures **(C, D)**. Each symbol represents one individual, and bars indicate geometric mean values. Symbols without any color represent participants who got infected before or after vaccination. Statistical analysis was performed between pre and post-vaccination using the Kruskal-Wallis test. ^*^P <0.05, ^**^P <0.01, ^***^P <0.001, ns, not significant; *P >*0.05.

In order to examine the dynamics of antibody responses pre- and post-administration of two primary doses and two booster doses of Covishield and/or mRNA (Moderna/Pfizer) vaccines, SARS-CoV-2 spike RBD-specific IgG antibody levels were examined at various time intervals. Notably, two doses of either Covishield or mRNA vaccine resulted in robust antibody responses initially (**Figures 1A, B**). However, after six months, IgG responses dropped significantly, reaching baseline levels in those with the primary Covishield vaccine (**Figure 1B**). Conversely, IgG responses decreased but remained significantly elevated for up to six months following the first dose of the mRNA vaccine (**Figure 1A**).

Upon receiving the first booster dose with the mRNA vaccine one year after the initial Covishield or mRNA doses, a significant recurrence of IgG antibodies was observed. This response declined by the sixth month after the first booster. Subsequently, we investigated the IgG responses once again after the second booster vaccination, administered one year after the first mRNA booster which was two years after the initial primary dose. Strikingly, we observed that the levels of RBD-specific IgG antibodies did not increase following the second booster vaccination with mRNA, regardless of primary vaccine type (**Figures 1A, B**). This decline in antibody responses was further supported by the decrease in antibody-fold rise (post vs. pre-booster 2, **Figures 1C, D**). Notably participants who had received four doses of mRNA as both primary and booster doses exhibited a greater reluctance in inducing antibody responses after booster doses (dose 3 and dose 4). A few participants (n=3-4) in each group had SARS-CoV-2 infection and no statistical differences were observed in the IgG levels between these two groups (**Supplementary Table 1**). The study highlights variations in antibody dynamics based on vaccine types and booster doses.

### 3.3 Dynamics of IgG subclasses in participants who received four doses of mRNA vaccines

**Figure 2:**
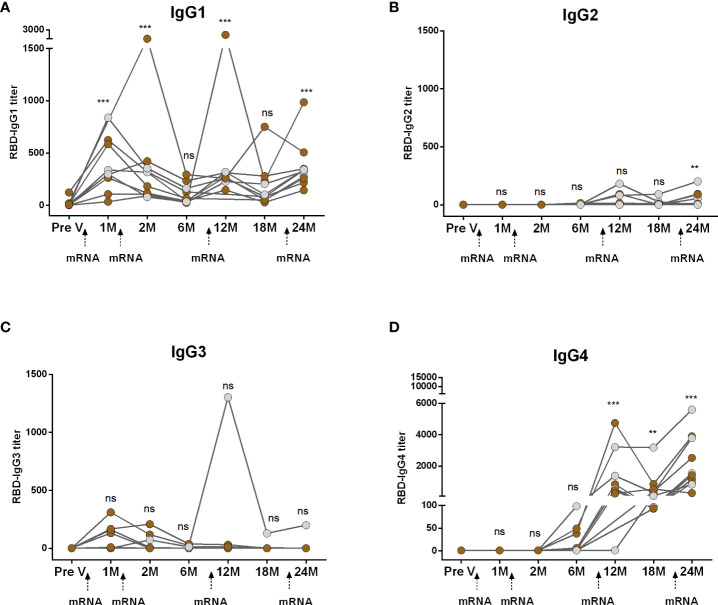
Kinetics of IgG subclasses after receiving COVID-19 mRNA vaccines as both primary and booster. RBD-specific IgG1 **(A)**, IgG2 **(B)**, IgG3 **(C)** and IgG4 **(D)** were analyzed in serum samples after four mRNA doses at different time intervals. Different doses of vaccine administration time points are indicated below the graphs. Samples were collected before vaccination (Pre-V), 1 month, 2/3 Months, 6 months, 12 months, 18 months, and 24 months after the first vaccination. Symbols without any color represent participants who got infected before or after vaccination. Statistical analysis was performed between pre and post-vaccination antibody titer using the Kruskal-Wallis test. **P <0.01, ***P <0.001, ns, not significant; *P* >0.05.

We conducted a comparative analysis of RBD-specific IgG subclass antibodies (IgG1, IgG2, IgG3, and IgG4) in participants who received two primary and two booster mRNA vaccine doses (**Figure 2**). Following both the first and second primary vaccinations, IgG1 emerged as the predominant subclass and remained significantly elevated for up to two months but declined to baseline levels within six months (**Figure 2A**). IgG2 responses were undetectable after primary vaccinations (**Figure 2B**). While a small number of participants displayed IgG3, responses were insignificant compared to pre-vaccine levels (**Figure 2C**). IgG4 antibodies were also undetectable initially but, surprisingly, IgG4 emerged within six months post primary mRNA doses (**Figure 2D**). Following boosters, IgG4 replaced IgG1 as the dominant subclass. After the first booster, IgG1 increased at one month but returned to baseline within six months (18 months from primary vaccination). The second booster prompted a slight IgG1 rise, although the responses were significantly higher than the baseline (**Figure 2A**). IgG2 responses increased after boosters. IgG3 responses were rare except in one case. IgG4 significantly rose significantly after both boosters (**Figure 2D**).

### 3.4 Dynamics of IgG subclasses in participants who received two Covishield and two mRNA vaccines

**Figure 3:**
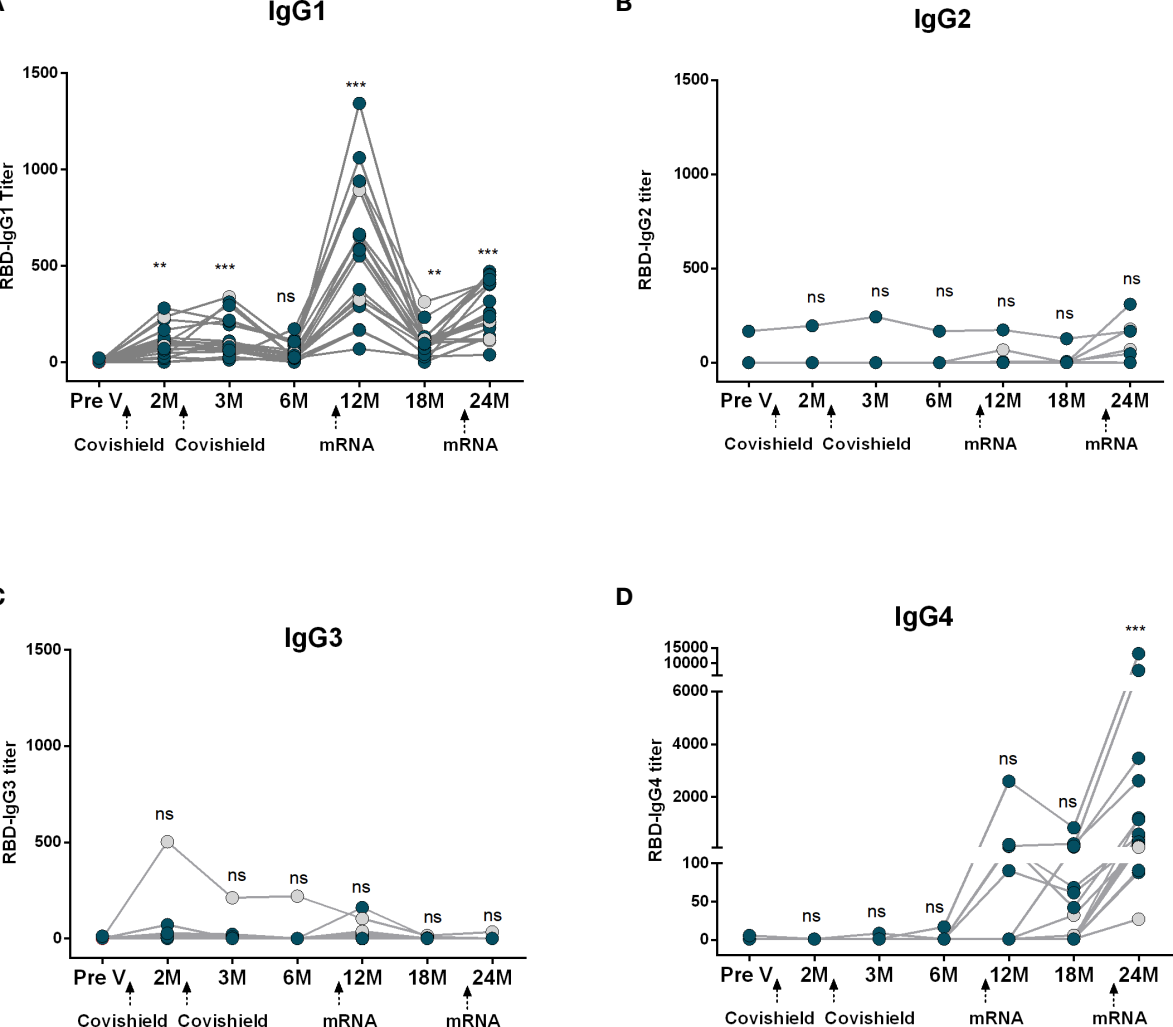
Kinetics of IgG subclasses after receiving COVID-19 vaccine Covishield as primary and mRNA as booster. RBD-specific IgG1 **(A)**, IgG2 **(B)**, IgG3 **(C)** and IgG4 **(D)** were analyzed in serum samples after two Covishield and two mRNA doses at different time intervals. Different doses of vaccine administration time points are indicated below the graphs. Samples were collected before vaccination (Pre-V), 1 month, 2/3 Months, 6 months, 12 months, 18 months, and 24 months after the first vaccination. Symbols without any color represent participants who got infected before or after vaccination. Statistical analysis was performed between pre and post-vaccination antibody titer using the Kruskal-Wallis test. **P <0.01, ***P <0.001, ns, not significant; *P* >0.05.

Next, we evaluated RBD-specific IgG subclass antibodies in participants who received two primary doses of Covishield followed by two mRNA booster doses (**Figure 3**). Prior to primary vaccination (Pre V), no IgG subclass responses were noted. Post-primary Covishield doses, IgG1 dominated for up to three months (**Figure 3A**), later declining to baseline within six months. IgG3 responses were insignificant (**Figure 3C**), while IgG2 and IgG4 responses were nearly absent until six months post-primary vaccination (**Figures 3B, D**).

Subsequently, we assessed the IgG subclass antibody responses following mRNA booster vaccination in the same group of participants who received Covishield primarily. Significant IgG1 surge occurred one month after the first booster, started to decline after six months but still remained higher than the baseline level. After the second booster IgG1 levels increased slightly, lower than the IgG1 levels found after the first booster dose (**Figure 3A**). IgG2 levels remained low after the first booster vaccine; however, a few participants exhibited increased IgG2 responses after the second booster (**Figure 3B**). Conversely, only a small number of participants showed IgG3 responses after the first booster, and no IgG3 responses were observed in any participants after the second booster dose (**Figure 3C**). Intriguingly, IgG4 was initially undetectable, rose 1-6 months post-first booster, significantly increasing after the second mRNA booster in all participants (**Figure 3D**).

### 3.5 Comparative analysis of IgG1 and IgG4 antibodies after booster vaccination

**Figure 4:**
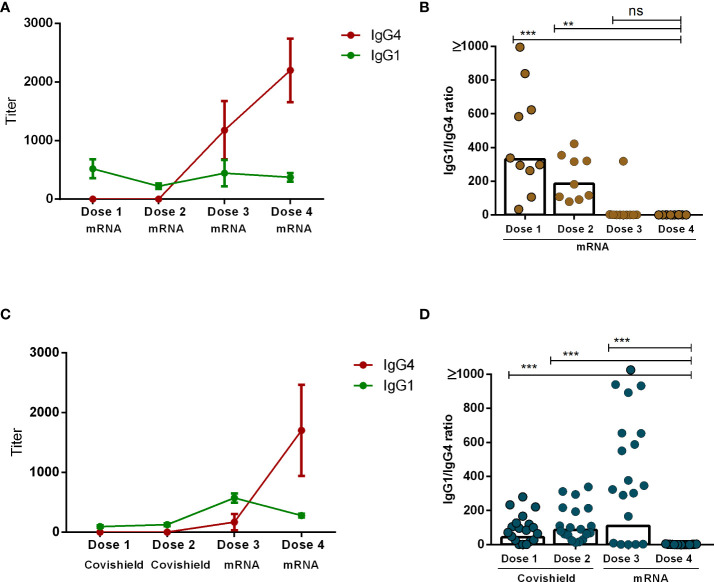
Comparison between IgG1 and IgG4 subclasses after four different doses of COVID-19 vaccines. RBD-specific IgG1 and IgG4 antibodies (mean+SEM) are shown in line graphs after four mRNA doses **(A)** and two primary Covishield and two booster mRNA doses **(C)**. IgG1 to IgG4 ratio was measured in individual participants one month after each vaccine dose are shown in graph **(B, D)**. Each symbol represents one individual, and bars indicate geometric mean values. Statistical analysis was performed between pre and post-vaccination antibody titer using the Kruskal-Wallis test. **P <0.01, ***P <0.001, ns, not significant; *P* >0.05.

Our analysis revealed that the principal subclasses of IgG antibodies present post-COVID-19 vaccination were either IgG1 or IgG4. The emergence of each subclass was dependent upon both the number of vaccine doses administered as well as the vaccine components. Specifically, IgG1 exhibited dominance one month subsequent to the administration of either the initial or second dose of both mRNA and Covishield vaccines. Conversely, IgG4 emerged as the predominant subclass following the administration of mRNA booster doses (dose 3 and dose 4) (**Figures 4A, C**). Next, we evaluated the IgG1 to IgG4 ratio one month after each vaccine dose on an individual level. Interestingly, IgG1 to IgG4 ratios decreased significantly as the number of vaccine doses increased. This phenomenon was more prominent in participants who received a total of four doses of the mRNA vaccine (**Figure 4B**). In contrast, within another subgroup of participants who initially received two doses of Covishield followed by two mRNA booster doses, the IgG1 to IgG4 ratio exhibited a significant decrease after the fourth dose (**Figure 4D**). We have performed a correlation test between serum IgG4 *vs*. IgG antibodies after combining all day points (1 month after each dose) after each vaccination in all participants (n=29) (**Supplementary Figure 1**). We have observed a significantly negative correlation (r=-0.47, P<0.0001) between IgG and IgG4, which may indicate that IgG4 levels negatively regulate overall antibody responses in serum after booster COVID-19 vaccination.

### 3.6 Serum anti-inflammatory cytokine level

We have determined anti-inflammatory cytokine IL-10 levels on after COVID-19 primary and booster doses. Participants who received mRNA as both primary and booster doses had increased levels of serum IL-10 levels after each vaccine doses (as determined by pre-vaccination *vs.* post-vaccination fold increase, **Supplementary Figure 2A**). No fold increase for IL-10 has been observed in participants received Covishield as primary doses (**Supplementary Figure 2B**). However, after receiving mRNA booster doses (dose 3 and dose 4, **Supplementary Figure 2B**), IL-10 levels increased in the serum specimens of the same participants. Based on these important findings, our results suggest that IL-10 cytokine may play a mechanistic role in the induction of IgG4 responses after booster COVID-19 vaccination.

## 4 Discussion

Understanding the distinctive attributes and long-lasting nature of the humoral immune response subsequent to primary and booster vaccinations holds paramount importance in optimizing vaccination strategies and evaluating immune reactions to emerging COVID-19 variants. This study investigated IgG antibody responses targeting SARS-CoV-2 spike RBD, encompassing four subclasses, over two years following ChAdOx1 nCoV-19 and/or mRNA vaccinations. To the best of our knowledge, this is the first study to compare the antibody responses of IgG subclasses subsequent to four different doses of Covishield and/or mRNA vaccines. This study revealed that primary vaccination induced IgG1 responses with limited IgG2 and IgG3, sustained until the initial booster. IgG4 responses showed a distinctive pattern. mRNA boosters triggered significant IgG4 surge, notably earlier and higher in mRNA-primary recipients. This research is part of a COVID-19 vaccination surveillance study being carried out in collaboration with the Government of Bangladesh, focusing exclusively on individuals who were administered four doses of COVID-19 vaccines. Due to the relatively low number of people in Bangladesh who received the fourth vaccine dose, the study participant was limited. A larger study will be needed representing diverse populations in different settings to confirm the induction of IgG4 subclasses and low immune responses after booster COVID-19 vaccination.

Our study demonstrated significant IgG antibody response enhancement after the third mRNA vaccine dose compared to primary vaccination in Bangladeshi adults. However, a differing trend emerged after the fourth mRNA booster dose given one year after the third dose, showing modest induction in IgG antibodies. This decline aligns with studies reporting waning immune responses after a fourth dose (5, 17). Three months after a fourth dose, a significant decay of IgG, IgA, and neutralizing antibodies was observed in participants received BNT162b2 vaccine in Israel (17). In another study, elderly individuals receiving a third dose showed declining specific antibodies against the Delta and Omicron variants over five months (18). This finding highlights the importance of continuous immune response monitoring, particularly in vulnerable groups, to refine vaccination strategies. The collective evidence suggests that repeated COVID-19 vaccinations might lead to waning immunity (5, 7, 18), prompting questions about optimal dosing schedules and long-term protection maintenance.

The dynamics of IgG responses to COVID-19 vaccines vary due to factors like age, prior exposure, and health conditions (6). Our study revealed IgG1 dominance after primary mRNA or Covishield vaccination, which is known for its robust antiviral activity and effector functions. Notably, spike RBD-specific IgG4 appears ~5-6 months after mRNA vaccination, consistent with other research (10). SARS-CoV-2 mRNA vaccination sustains mRNA/spike antigens in blood and lymph nodes (19); spike protein was found in post-COVID-19 mRNA vaccine myocarditis (20). Additionally, another classic research showed IgG4 emerges with prolonged antigen exposure. Beekeepers displayed increasing bee venom IgG4 over seasons, shifting from IgG1 dominance to IgG4 prevalence after 6 months (21). Our study aligns with previous research, showing slow kinetics of IgG4 appearance in the blood, taking several months after mRNA vaccination. However, the reason why the adenoviral vector-based Covishield vaccine did not enhance IgG4 responses is not entirely clear. It is possible that the level of RBD expression might not have reached a threshold sufficient to induce IgG4 switching.

The role of the least abundant IgG4 is not well understood in terms of its regulatory functions in various diseases. Depending on the type and condition of the disease, this regulatory role can be either beneficial or detrimental (11). IgG4 shows an advantageous effect in response to allergen and parasitic infection allergen and parasitic response while countering hypersensitivity (22–24), yet contributing to the onset and progression of autoimmune diseases, tumor immunology, and IgG4-related diseases, lacking specific treatments (11). Certain autoimmune skin diseases severity correlates with antigen-specific IgG4 levels (25, 26). In severe COVID-19, higher RBD-specific IgG4 levels were noted in deceased individuals than survivors, potentially due to cytokine storms including both anti- and pro-inflammatory cytokines, including IL-10, which could further driving IgG4 induction (12). Class switching to IgG4 subclass antibody is mostly associated with T helper 2 cell responses (11). In concert with other regulatory molecules, IL-10 plays an important role in the induction of IgG4 subclass (11, 27). IL-10 is known as an anti-inflammatory cytokine in preventing inflammation and can hamper host response to microbial pathogenesis (28). After observing increased IgG4 responses after booster vaccination, our investigation focused on exploring IL-10 induction following Covishield and mRNA vaccination. Participants who received mRNA for both primary and booster doses exhibited elevated serum IL-10 levels after each vaccine administration. This is in line with our findings that IgG4 antibody levels in the serum began to rise 6 months post-primary mRNA vaccination and continued to increase following the boosters. Our observation of increased IL-10 after each dose of mRNA vaccination suggest long-time induction of IL-10 might produce stimulatory signals favorable to induce IgG4 class switching after mRNA vaccination. Interestingly, participants who received Covishield as primary vaccines, no increase of IL-10 or IgG4 antibody responses was observed from the baseline. However, when the same participants received mRNA as booster doses, both IL-10 and IgG4 increased. Induction of IL-10 as well as IgG4 might be the result of long term presence of mRNA/spike antigens in blood and lymph nodes after SARS-CoV-2 mRNA vaccination (19). This is consistent to the results showing that extended immunization of mice with SARS-CoV-2 RBD booster vaccination induced IL-10 responses which overall induced humoral and cellular immunological tolerance in mice (29). Based on these crucial findings, our results suggest that IL-10 cytokine may mechanistically contribute to the induction of IgG4 responses following booster COVID-19 vaccination.

IgG4 related diseases also reported in some case reports after receiving COVID-19 mRNA vaccine. For example, two weeks after receiving two doses of mRNA vaccine, IgG4-related lung disease developed in a 71-year-old man (30) and IgG4-related nephritis relapsed in a 66-year-old individual following vaccination (31). Several investigations have found that COVID-19 vaccination is associated with the development of autoimmune diseases (32, 33). Additionally, increased levels of IgG have been found in SARS-CoV-2 breakthrough infections. However, IgG4 has limited ability to activate antibody-dependent immune effector mechanisms due to its unique structural differences from other IgG subclasses resulting from Fab-arm exchange and a greater propensity for glycosylation in the variable domains (34, 35). This suggests that COVID-19-vaccinated individuals with elevated IgG4 levels may have a higher risk of breakthrough infections, as SARS-CoV-2 employs various evasion strategies to evade immune surveillance and counterattack through the suppression of interferon production (36, 37), disruption of antigen presentation (38), and induction of lymphopenia through the formation of syncytia (39). Overall, Elevated IgG4 might alter vaccine efficacy or trigger anti-inflammatory responses. Further research is needed to fully understand its regulatory functions and its implications in SARS-CoV-2 breakthrough infection as well as vaccination. Future vaccination strategies need to be designed using thorough research data, requiring larger studies that encompass diverse populations across different settings. This is crucial to prevent immunological tolerance and the induction of IgG4 subclasses after administering booster COVID-19 vaccinations using various regimens. Additionally, it involves optimizing time intervals between doses.

## Data availability statement

The raw data supporting the conclusions of this article will be made available by the authors, without undue reservation.

## Ethics statement

The studies involving humans were approved by Ethical Review Committee (ERC) of icddr,b. The studies were conducted in accordance with the local legislation and institutional requirements. The participants provided their written informed consent to participate in this study.

## Author contributions

MA: Conceptualization, Data curation, Formal analysis, Investigation, Methodology, Software, Supervision, Validation, Writing – original draft, Writing – review & editing. MRI: Data curation, Methodology, Validation, Writing – original draft. FKhat: Data curation, Investigation, Methodology, Supervision, Writing – review & editing. US: Investigation, Methodology, Writing – review & editing. SJ: Investigation, Methodology, Writing – review & editing. SR: Methodology, Writing – review & editing. IT: Methodology, Writing – review & editing, Resources, Supervision. TA: Methodology, Resources, Supervision, Writing – review & editing. IK: Methodology, Supervision, Writing – review & editing. AA: Methodology, Supervision, Writing – review & editing, Funding acquisition, Resources. ZK: Methodology, Supervision, Writing – review & editing. MTI: Methodology, Supervision, Writing – review & editing, Project administration. FKha: Writing – review & editing. PB: Writing – review & editing, Data curation. FA: Data curation, Writing – review & editing. SA: Writing – review & editing. MMR: Writing – review & editing. MH: Writing – review & editing. ANA: Writing – review & editing. ASMA: Writing – review & editing. MR: Writing – review & editing. ER: Writing – review & editing. JH: Writing – review & editing. RL: Writing – review & editing. MF: Writing – review & editing. FC: Writing – review & editing. AK: Supervision, Writing – review & editing. SB: Funding acquisition, Resources, Writing – review & editing. TS: Project administration, Writing – review & editing. TB: Conceptualization, Formal analysis, Funding acquisition, Investigation, Methodology, Project administration, Resources, Supervision, Validation, Writing – review & editing. FQ: Funding acquisition, Project administration, Resources, Supervision, Writing – review & editing.

## References

[1] COVID-19 Vaccine Tracker (2023). Available at: https://covid19.trackvaccines.org/country/Bangladesh/.

[2] Rahmani K Shavaleh R Forouhi M Disfani HF Kamandi M Oskooi RK. The effectiveness of COVID-19 vaccines in reducing the incidence, hospitalization, and mortality from COVID-19: A systematic review and meta-analysis. Front Public Health (2022) 10:873596. doi: 10.3389/fpubh.2022.873596 36091533 PMC9459165

[3] Khanam F Islam MT Ahmmed F Ahmed SU Hossen MI Rajib MH. Measuring the effectiveness of COVID-19 vaccines used during a surge of the delta variant of SARS-CoV-2 in Bangladesh: A test-negative design evaluation. Vaccines (2022) 10(12):2069. doi: 10.3390/vaccines10122069 36560479 PMC9780914

[4] Andrews N Stowe J Kirsebom F Toffa S Sachdeva R Gower C. Effectiveness of COVID-19 booster vaccines against COVID-19-related symptoms, hospitalization and death in England. Nat Med (2022) 28(4):831–7. doi: 10.1038/s41591-022-01699-1 PMC9018410 35045566

[5] Menni C May A Polidori L Louca P Wolf J Capdevila J. COVID-19 vaccine waning and effectiveness and side-effects of boosters: a prospective community study from the ZOE COVID Study. Lancet Infect Dis (2022) 22(7):1002–10. doi: 10.1016/s1473-3099(22)00146-3 PMC8993156 35405090

[6] Bhuiyan TR Akhtar M Khaton F Rahman SIA Ferdous J Alamgir ASM. Covishield vaccine induces robust immune responses in Bangladeshi adults. IJID Regions (Online) (2022) 3:211–7. doi: 10.1016/j.ijregi.2022.04.006 PMC9050186 35720155

[7] Goh YS Rouers A Fong SW Zhuo NZ Hor PX Loh CY. Waning of specific antibodies against Delta and Omicron variants five months after a third dose of BNT162b2 SARS-CoV-2 vaccine in elderly individuals. Front Immunol (2022) 13:1031852. doi: 10.3389/fimmu.2022.1031852 36451833 PMC9704817

[8] Seow J Graham C Merrick B Acors S Pickering S Steel KJA. Longitudinal observation and decline of neutralizing antibody responses in the three months following SARS-CoV-2 infection in humans. Nat Microbiol (2020) 5(12):1598–607. doi: 10.1038/s41564-020-00813-8 PMC7610833 33106674

[9] Subramanian SV Kumar A. Increases in COVID-19 are unrelated to levels of vaccination across 68 countries and 2947 counties in the United States. Eur J Epidemiol (2021) 36(12):1237–40. doi: 10.1007/s10654-021-00808-7 PMC8481107 34591202

[10] Irrgang P Gerling J Kocher K Lapuente D Steininger P Habenicht K. Class switch toward noninflammatory, spike-specific IgG4 antibodies after repeated SARS-CoV-2 mRNA vaccination. Sci Immunol (2023) 8(79):eade2798. doi: 10.1126/sciimmunol.ade2798 36548397 PMC9847566

[11] Rispens T Huijbers MG. The unique properties of IgG4 and its roles in health and disease. Nat Rev Immunol (2023) 1-16:763–78. doi: 10.1038/s41577-023-00871-z PMC10123589 37095254

[12] Moura AD da Costa HHM Correa VA de SLAK Lindoso JAL De Gaspari E. Assessment of avidity related to IgG subclasses in SARS-CoV-2 Brazilian infected patients. Sci Rep (2021) 11(1):17642. doi: 10.1038/s41598-021-95045-z 34480056 PMC8417219

[13] Della-Torre E Lanzillotta M Strollo M Ramirez GA Dagna L Tresoldi M. Serum IgG4 level predicts COVID-19 related mortality. Eur J Internal Med (2021) 93:107–9. doi: 10.1016/j.ejim.2021.09.012 PMC8461218 34598853

[14] Buhre JS Pongracz T Künsting I Lixenfeld AS Wang W Nouta J. mRNA vaccines against SARS-CoV-2 induce comparably low long-term IgG Fc galactosylation and sialylation levels but increasing long-term IgG4 responses compared to an adenovirus-based vaccine. Front Immunol (2022) 13:1020844. doi: 10.3389/fimmu.2022.1020844 36713457 PMC9877300

[15] Bhuiyan TR Akhtar M Akter A Khaton F Rahman SIA Ferdous J. Seroprevalence of SARS-CoV-2 antibodies in Bangladesh related to novel coronavirus infection. IJID Regions (2022) 2:198–203. doi: 10.1016/j.ijregi.2022.01.013 35721426 PMC8809641

[16] Akhtar M Basher SR Nizam NN Kamruzzaman M Khaton F Banna HA. Longevity of memory B cells and antibodies, as well as the polarization of effector memory helper T cells, are associated with disease severity in patients with COVID-19 in Bangladesh. Front Immunol (2022) 13:1052374. doi: 10.3389/fimmu.2022.1052374 36578502 PMC9791541

[17] Canetti M Barda N Gilboa M Indenbaum V Mandelboim M Gonen T. Immunogenicity and efficacy of fourth BNT162b2 and mRNA1273 COVID-19 vaccine doses; three months follow-up. Nat Commun (2022) 13(1):7711. doi: 10.1038/s41467-022-35480-2 36513665 PMC9745767

[18] Renia L Goh YS Rouers A Le Bert N Chia WN Chavatte JM. Lower vaccine-acquired immunity in the elderly population following two-dose BNT162b2 vaccination is alleviated by a third vaccine dose. Nat Commun (2022) 13(1):4615. doi: 10.1038/s41467-022-32312-1 35941158 PMC9358634

[19] Röltgen K Nielsen SCA Silva O Younes SF Zaslavsky M Costales C. Immune imprinting, breadth of variant recognition, and germinal center response in human SARS-CoV-2 infection and vaccination. Cell (2022) 185(6):1025–40.e14. doi: 10.1016/j.cell.2022.01.018 35148837 PMC8786601

[20] Yonker LM Swank Z Bartsch YC Burns MD Kane A Boribong BP. Circulating spike protein detected in post-COVID-19 mRNA vaccine myocarditis. Circulation (2023) 147(11):867–76. doi: 10.1161/circulationaha.122.061025 PMC10010667 36597886

[21] Aalberse RC van der Gaag R van Leeuwen J. Serologic aspects of IgG4 antibodies. I. Prolonged immunization results in an IgG4-restricted response. J Immunol (Baltimore Md: 1950) (1983) 130(2):722–6. 6600252

[22] Kurniawan A Yazdanbakhsh M van Ree R Aalberse R Selkirk ME Partono F. Differential expression of IgE and IgG4 specific antibody responses in asymptomatic and chronic human filariasis. J Immunol (Baltimore Md: 1950) (1993) 150(9):3941–50. doi: 10.4049/jimmunol.150.9.3941 8473742

[23] Holgate ST Polosa R. Treatment strategies for allergy and asthma. Nat Rev Immunol (2008) 8(3):218–30. doi: 10.1038/nri2262 18274559

[24] Akdis CA. Therapies for allergic inflammation: refining strategies to induce tolerance. Nat Med (2012) 18(5):736–49. doi: 10.1038/nm.2754 22561837

[25] Huijbers MG Vink AF Niks EH Westhuis RH van Zwet EW de Meel RH. Longitudinal epitope mapping in MuSK myasthenia gravis: implications for disease severity. J neuroimmunol (2016) 291:82–8. doi: 10.1016/j.jneuroim.2015.12.016 26857500

[26] Futei Y Amagai M Ishii K Kuroda-Kinoshita K Ohya K Nishikawa T. Predominant IgG4 subclass in autoantibodies of pemphigus vulgaris and foliaceus. J Dermatol Sci (2001) 26(1):55–61. doi: 10.1016/s0923-1811(00)00158-4 11323221

[27] Trampert DC Hubers LM van de Graaf SFJ Beuers U. On the role of IgG4 in inflammatory conditions: lessons for IgG4-related disease. Biochim Biophys Acta Mol basis Dis (2018) 1864(4 Pt B):1401–9. doi: 10.1016/j.bbadis.2017.07.038 28782655

[28] Iyer SS Cheng G. Role of interleukin 10 transcriptional regulation in inflammation and autoimmune disease. Crit Rev Immunol (2012) 32(1):23–63. doi: 10.1615/critrevimmunol.v32.i1.30 22428854 PMC3410706

[29] Gao FX Wu RX Shen MY Huang JJ Li TT Hu C. Extended SARS-CoV-2 RBD booster vaccination induces humoral and cellular immune tolerance in mice. iScience (2022) 25(12):105479. doi: 10.1016/j.isci.2022.105479 36338436 PMC9625849

[30] Tasnim S Al-Jobory O Hallak A Bharadwaj T Patel M. IgG4 related pleural disease: Recurrent pleural effusion after COVID-19 vaccination. Respirol Case Rep (2022) 10(10):e01026. doi: 10.1002/rcr2.1026 36187460 PMC9483611

[31] Masset C Kervella D Kandel-Aznar C Fantou A Blancho G Hamidou M. Relapse of IgG4-related nephritis following mRNA COVID-19 vaccine. Kidney Int (2021) 100(2):465–6. doi: 10.1016/j.kint.2021.06.002 PMC8186934 34116086

[32] Gadi SRV Brunker PAR Al-Samkari H Sykes DB Saff RR Lo J. Severe autoimmune hemolytic anemia following receipt of SARS-CoV-2 mRNA vaccine. Transfusion (2021) 61(11):3267–71. doi: 10.1111/trf.16672 PMC8661722 34549821

[33] Portuguese AJ Sunga C Kruse-Jarres R Gernsheimer T Abkowitz J. Autoimmune- and complement-mediated hematologic condition recrudescence following SARS-CoV-2 vaccination. Blood Adv (2021) 5(13):2794–8. doi: 10.1182/bloodadvances.2021004957 PMC8276576 34255033

[34] Lighaam LC Rispens T. The immunobiology of immunoglobulin G4. Semin liver Dis (2016) 36(3):200–15. doi: 10.1055/s-0036-1584322 27466791

[35] Vidarsson G Dekkers G Rispens T. IgG subclasses and allotypes: from structure to effector functions. Front Immunol (2014) 5:520. doi: 10.3389/fimmu.2014.00520 25368619 PMC4202688

[36] Oh SJ Shin OS. SARS-CoV-2-mediated evasion strategies for antiviral interferon pathways. J Microbiol (Seoul Korea) (2022) 60(3):290–9. doi: 10.1007/s12275-022-1525-1 PMC8817151 35122601

[37] Xia H Cao Z Xie X Zhang X Chen JY Wang H. Evasion of type I interferon by SARS-Cov-2. Cell Rep (2020) 33(1):108234. doi: 10.1016/j.celrep.2020.108234 32979938 PMC7501843

[38] Yoo JS Sasaki M Cho SX Kasuga Y Zhu B Ouda R. SARS-CoV-2 inhibits induction of the MHC class I pathway by targeting the STAT1-IRF1-NLRC5 axis. Nat Commun (2021) 12(1):6602. doi: 10.1038/s41467-021-26910-8 34782627 PMC8594428

[39] Lin L Li Q Wang Y Shi Y. Syncytia formation during SARS-CoV-2 lung infection: a disastrous unity to eliminate lymphocytes. Cell Death Differ (2021) 28(6):2019–21. doi: 10.1038/s41418-021-00795-y PMC8114657 33981020

