# Supplementary material for: Appearance of tolerance-induction and non-inflammatory SARS-CoV-2 spike-specific IgG4 antibodies after COVID-19 booster vaccinations
Source: Front Immunol. 2023 Dec 20;14:1309997. doi: 10.3389/fimmu.2023.1309997 (PMC10763240; doi:10.3389/fimmu.2023.1309997)
Supplement: Supplementary file 1 [file Presentation_1.pptx]

## Slide 1
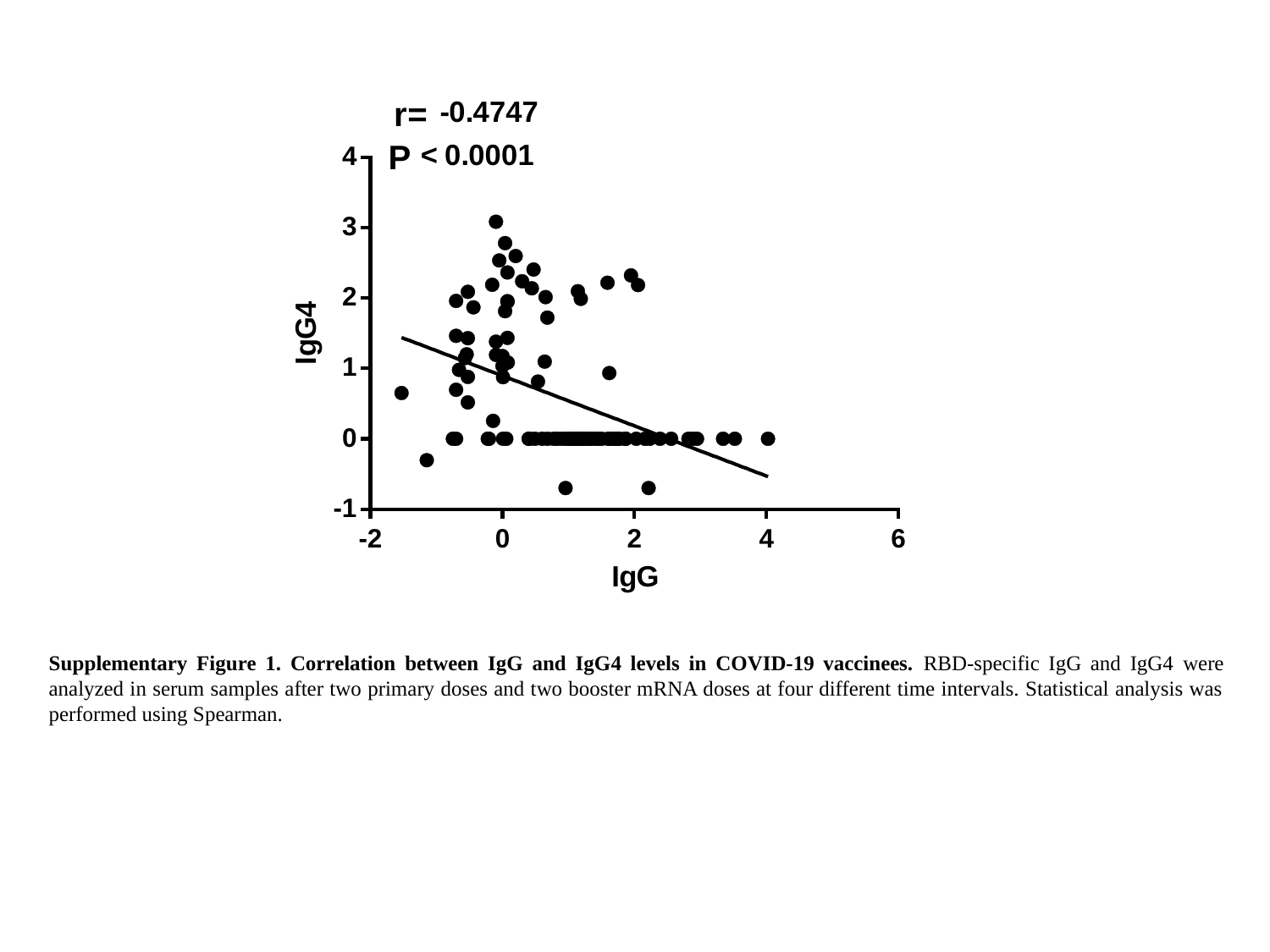

Supplementary Figure 1. Correlation between IgG and IgG4 levels in COVID-19 vaccinees. RBD-specific IgG and IgG4 were analyzed in serum samples after two primary doses and two booster mRNA doses at four different time intervals. Statistical analysis was performed using Spearman.

## Slide 2
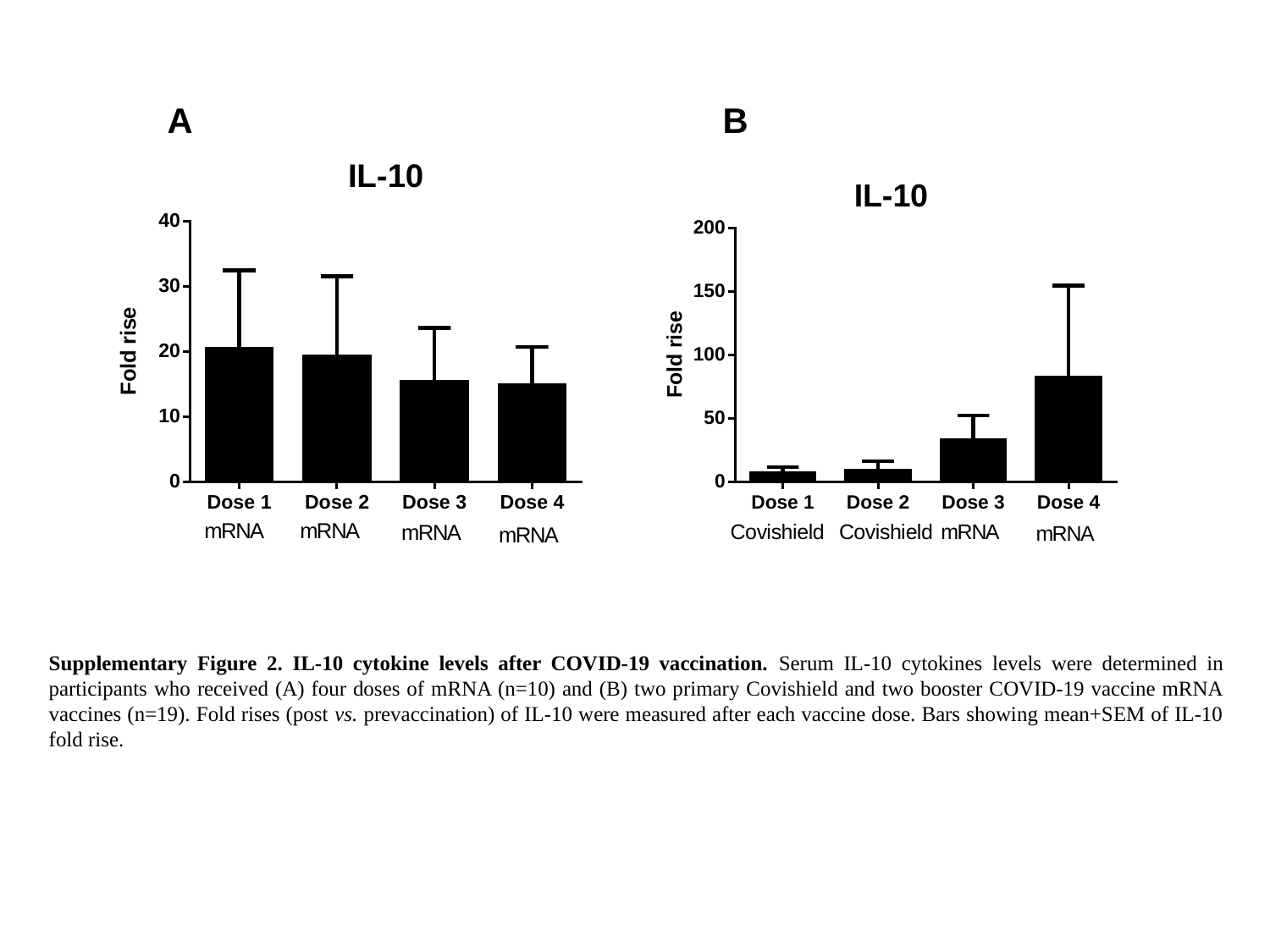

Supplementary Figure 2. IL-10 cytokine levels after COVID-19 vaccination. Serum IL-10 cytokines levels were determined in participants who received (A) four doses of mRNA (n=10) and (B) two primary Covishield and two booster COVID-19 vaccine mRNA vaccines (n=19). Fold rises (post vs. prevaccination) of IL-10 were measured after each vaccine dose. Bars showing mean+SEM of IL-10 fold rise.
