# Supplementary material for: Appearance of tolerance-induction and non-inflammatory SARS-CoV-2 spike-specific IgG4 antibodies after COVID-19 booster vaccinations
Source: Front Immunol. 2023 Dec 20;14:1309997. doi: 10.3389/fimmu.2023.1309997 (PMC10763240; doi:10.3389/fimmu.2023.1309997)
Supplement: Supplementary file 2 [file Table_1.docx]

|  | **Infected** | **Uninfected** |  | **Infected** | **Uninfected** |  | **Infected** | **Uninfected** |  |
| --- | --- | --- | --- | --- | --- | --- | --- | --- | --- |
|  | **IgG** | **IgG** | **P value** | **IgG1** | **IgG1** | **P value** | **IgG4** | **IgG4** | **P value** |
| **Group**  **A** |  |  |  |  |  |  |  |  |  |
| Pre | 246.8 | 1123.2 | 0.200 | 1.0 | 8.6 | 0.105 | 1.0 | 1.0 | 1.00 |
| 1M | 20471.2 | 16940.0 | 0.517 | 231.5 | 418.8 | 0.829 | 1.0 | 1.0 | 1.00 |
| 2M | 22515.0 | 21130.9 | 0.867 | 168.6 | 198.6 | 0.524 | 1.0 | 1.0 | 1.00 |
| 6M | 13588.1 | 11077.8 | 0.667 | 50.2 | 91.9 | 0.448 | 12.7 | 3.3 | 0.27 |
| 12M | 18315.0 | 62221.6 | 0.111 | 267.1 | 251.9 | 0.476 | 222.9 | 457.5 | 0.75 |
| 18M | 7628.2 | 9993.1 | 0.562 | 96.5 | 133.7 | 0.562 | 321.8 | 230.3 | 0.61 |
| 24M | 5446.4 | 4399.2 | 0.829 | 318.6 | 334.4 | 1.000 | 2293.2 | 1271.9 | 0.48 |
| **Group B** |  |  |  |  |  |  |  |  |  |
| Pre | 77.5 | 798.8 | 0.147 | 1.0 | 1.5 | 1.000 | 1.0 | 1.1 | 1.00 |
| 1M | 7357.2 | 8040.5 | 0.899 | 24.8 | 49.4 | 0.596 | 1.0 | 1.0 | 1.00 |
| 2M | 21354.9 | 22971.1 | 0.899 | 95.7 | 82.8 | 0.665 | 1.0 | 1.2 | 1.00 |
| 6M | 6021.2 | 3794.2 | 0.411 | 65.2 | 23.0 | 0.097 | 1.0 | 1.2 | 1.00 |
| 12M | 68784.3 | 29341.0 | 0.307 | 552.8 | 440.6 | 0.736 | 1.0 | 6.3 | 0.39 |
| 18M | 14901.7 | 8875.4 | 0.262 | 137.3 | 48.7 | 0.147 | 3.7 | 9.7 | 0.55 |
| 24M | 9110.4 | 6402.8 | 0.597 | 184.6 | 254.7 | 0.238 | 113.8 | 653.0 | 0.07 |

**Supplementary Table 1. Antibody levels in SARS-CoV-2 infected and uninfected participants.**
